# Crashworthiness Assessment of Carbon/Glass Epoxy Hybrid Composite Tubes Subjected to Axial Loads

**DOI:** 10.3390/polym14194083

**Published:** 2022-09-29

**Authors:** Ali Farokhi Nejad, Seyd Saied Rahimian Koloor, Mohd Luqman Hakim Arifin, Ali Shafiei, Shukur Abu Hassan, Mohd Yazid Yahya

**Affiliations:** 1 Department of Mechanical and Aerospace Engineering, Politecnico di Torino, 10129 Turin, Italy; 2 School of Mechanical Engineering, Faculty of Engineering, University Technology Malaysia, Johor Bahru 81310, Malaysia; 3 Institute for Nanomaterials, Advanced Technologies and Innovation (CXI), Technical University of Liberec (TUL), Studentska 2, 461 17 Liberec, Czech Republic; 4 Department of Aerospace Engineering, Faculty of Engineering, University Putra Malaysia, Kuala Lumpur 43400, Malaysia; 5 Department of Mechanical Engineering, AMICI R&D Group, Tehran 1474585745, Iran; 6 Centre for Advanced Composite Materials (CACM), University Technology Malaysia, Johor Bahru 81310, Malaysia

**Keywords:** hybrid composites, composite tube, crashworthiness, finite element model, energy absorption, axial load

## Abstract

The crashworthiness of composite tubes is widely examined for various types of FRP composites. However, the use of hybrid composites potentially enhances the material characteristics under impact loading. In this regard, this study used a combination of unidirectional glass–carbon fibre reinforced epoxy resin as the hybrid composite tube fabricated by the pultrusion method. Five tubes with different length aspect ratios were fabricated and tested, in which the results demonstrate “how structural energy absorption affects by increasing the length of tubes”. Crash force efficiency was used as the criterion to show that the selected L/D are acceptable of crash resistance with 95% efficiency. Different chamfering shapes as the trigger mechanism were applied to the tubes and the triggering effect was examined to understand the impact capacity of different tubes. A finite element model was developed to evaluate different crashworthiness indicators of the test. The results were validated through a good agreement between experimental and numerical simulations. The experimental and numerical results show that hybrid glass/carbon tubes accomplish an average 25.34 kJ/kg specific energy absorption, average 1.43 kJ energy absorption, average 32.43 kN maximum peak load, and average 96.67% crash force efficiency under quasi-static axial loading. The results show that selecting the optimum trigger mechanism causes progressive collapse and increases the specific energy absorption by more than 35%.

## 1. Introduction

Improvement of reliability and crashworthiness of motorized vehicles in advanced industries such as automotive, aeronautic, naval, etc., has been a big challenge for manufacturers. Thin-walled tubes were utilized as one of the best options to absorb the impact energy during a collision [1]. By the means of cost-effective tube products, various crash boxes have been made of available metals in the market, e.g., steel and aluminium alloys [2]. However, the most common production method for crash box tubes is a metal stamping and a tailored welding process that is made by several spot welding points [3]. These points make a heat-affected zone area during the manufacturing process and over time, those areas are degraded gradually [4]. Therefore, at the crash scene, the spot-welded area will fail and it would be a source of additional damage to the passengers. Furthermore, many sectors, due to ecological issues such as global warming, air pollution, and reduction of CO_2_ emission production of lightweight structures have been paid attention to [5].

Several studies have been carried out to enhance the impact resistance capability of composite structures subjected to different strain rates [6,7,8]. Some researchers show that designing appropriate composite laminates will lead to a composite tube with progressive collapse capability that makes it better than conventional metallic tubes in terms of specific energy absorption (SEA) [8,9,10,11]. Moreover, the manufacturing process of composite tubes is much easier than the metal forming process. Although many investigations have been carried out on the improvement of the crashworthiness of composite tubes, less attention has been paid to the high price of raw materials and manufacturing costs [12,13].

Hybrid materials with diverse properties combined with the main components, provide the perfect synergy of properties that enhance the structural behaviour and lead to an end product with superb features [14,15]. In fibrous composites, fibre hybridization is a way to improve material properties and general toughness, in which two or more fibres are combined as a reinforcement in the polymer matrix [6,16]. Different kinds of synthetic fibres have been made for several applications. Aramid/Kevlar is one of the best fibres for composite structures under high-impact loading. It has been widely used in military applications such as bulletproof vests and helmets. However, Aramid/Kevlar fibres are weak against humidity and UV radiation. Moreover, using Kevlar for general applications, e.g., passenger’s car crash boxes is not a good choice regarding availability and cost [6]. Carbon fibre is a well-known synthetic fibre that has excellent mechanical and electrical conductivity, high fatigue strength, and corrosion resistance. On the other hand, CFRPs are expensive and brittle under impact loading. The other famous synthetic fibre has good mechanical properties at a low cost; however, its fatigue properties and corrosion resistance are not acceptable for long-term services. Therefore, the combination of GFRP and CFRP could be an option to enhance the material properties of a hybrid structure under impact loading. Much research has been conducted in the use of natural fibres but these do not possess enough strength for impact resistance and often natural fibres were combined with synthetic fibres as the second filler. In these cases, some good achievements were obtained in reducing the cost of composite structures subjected to low-impact loading. Some studies have been conducted in the use of hybrid fibre composites to generate thin-walled FRP tubes and evaluate the crashworthiness of different geometries and cross sections [17,18]. In some research, the failure modes on crash boxes were investigated experimentally and it has been found that the general failure modes on composite tubes consist of delamination, bending, axial cracking, and fibre fracturing [8,19,20,21,22].

Based on previous literature, the failure mechanism of hybrid fibre structures under axial loading has different failure modes, i.e., mode I, mode II, and mixed mode [23,24]. However, the different failure mechanisms are a key factor in raising the energy absorption value. Moreover, numerous studies have been conducted on the collapse behaviour of thin-walled composite tubes [13,25]. The layer configuration, e.g., fibre angle, or layer stacking effects the dynamic responses of structures. Due to the fact that the failure mechanism and progressive collapse behaviour are the key points in increasing the crash resistance of FRP thin-walled tubes, the edge chamfer trigger forms at the tip of crash boxes were utilized to provide a progressive collapse behaviour during the crash, and finally increases the energy absorption of the structure [26]. Some studies have been performed on the effect of different materials on the energy absorption of similar geometry [27]. Moreover, they have shown that the trigger mechanism changes the failure mode from catastrophic failure to the progressive collapse mode [28,29,30]. The buckling of a thin-walled tube is highly dependent on the aspect ratio of the tubes [31]. Many researchers have studied the effect of aspect ratio (length to diameter (L/D) and thickness to diameter (t/D)) of composite tubes for crashworthiness assessment [32,33]. However, fewer studies have concentrated on the hybrid composite tube.

Thanks to advances in numerical methods including the finite element method (FE), many studies have been carried out on different aspects of the crashworthiness assessment of thin-walled structures under axial loading and subjected to different strain rates. Some researchers have evaluated the crashworthiness indicators such as specific energy absorption, energy absorption, maximum peak load, and mean crush load [34]. Moreover, different damage mechanisms were reported by previous literature [35,36]. Unlike isotropic materials, the FRP composite tubes are made with different fibre volume fractions, and it makes it more complex to predict the behaviour of a different component. Prediction of the damage mechanism of a composite structure is a difficult procedure and it requires several experimental tests to predict the structural behaviour after failure [37,38]. For time and cost reduction, the FE method is a vital tool to realize the full-scale model behaviour before the production stage. Many models are available for different materials, such as conventional FRP composite and some advanced materials such as auxetic materials, 2D lattice structures, and auxetic foam as the core of sandwich panels [39,40,41]. However, there are limited numerical models to predict the dynamic behaviour of hybrid fibre polymer matrix composites. However, through a numerical model, optimization of thin-walled tubes is possible. Some researchers have proposed shape and topology optimization methods to determine an optimum geometry against a specific loading condition [42]. Moreover, the statistical method was used to apply the design of the experimental method to minimize the number of experiments and find the significant factors in an experimental test or numerical simulation.

This study uses experimental and computational approaches to investigate the mechanical performance of hybrid carbon/glass/epoxy composite tubes subjected to monotonic loading. The structural behaviour, nonlinear response, failure modes, and mechanisms, as well as the crashworthiness indicators of the composite tubes with various aspect ratios, were examined. Furthermore, the effect of the trigger mechanism was evaluated. An FE model was developed and validated by examining the result with the experiment counterpart.

## 2. Materials and Methods

### 2.1. Experimental Procedure

**Figure 1:**
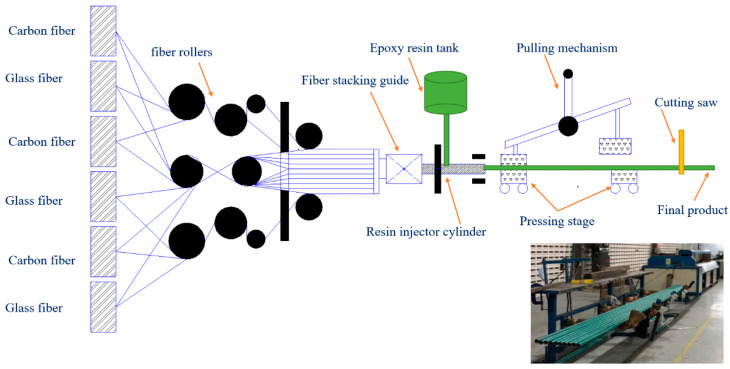
Schematic view of pultrusion processing method.

In this study, a hybrid composite tube with a combination of carbon fibre and glass fibre with a 30 ± 2% volume fraction of each kind was used as the filler for an epoxy resin. The inner diameter of all the samples was considered to equal 34 mm and the thickness of the tubes was kept at 2 ± 0.1 mm. The tubes were made of 16 layers consisting of a combination of carbon and glass fibres. The volume fraction of the matrix was 40% and the rest was made up of the fibres. In this study, the volume fraction of fibres has an equal value for GFRP and CFRP of 30%. The composite tubes have unidirectional fibres, and the tubes were made through the pultrusion process. The pultrusion machine can make composite tubes with single or various kinds of fibres. Moreover, by changing the extruder die cross section, different cross sections and diameters can be manufactured. However, investigation of the effect of different fibre volume fractions and different layer configurations can be examined in future studies. The final product from the pultrusion machine is the composite pipe of 3m length. The tubes can be sized by a diamond saw at a specific length. The burn test was applied to five random samples to verify the repeatability of fibre volume fraction. Figure 1 shows the schematic view of the pultrusion processing method.

**Figure 2:**
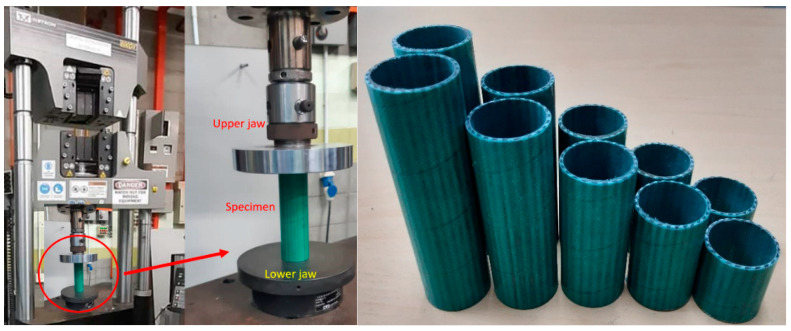
Setup of experiment for compression loading test.

The samples were cut in 5 different aspect ratios, i.e., 1, 1.5, 2, 2.5, and 3. To avoid early-stage failure, it should be considered that the upper and lower surfaces should be completely flat. The samples were used in an INSTRON universal testing machine with 100 kN load cell capacity. The loading rate was set as 5 mm/min. For each test, five samples were prepared. In this study, the thickness and hoop diameters were kept constant and only the length variation was examined. The setup of the experiment and tube sample with different aspect ratios were shown in Figure 2. Setup of experiment and tube sample with different aspect ratios. Table 1 shows some general information about the fibres and matrices used in this study.

**Figure 3:**
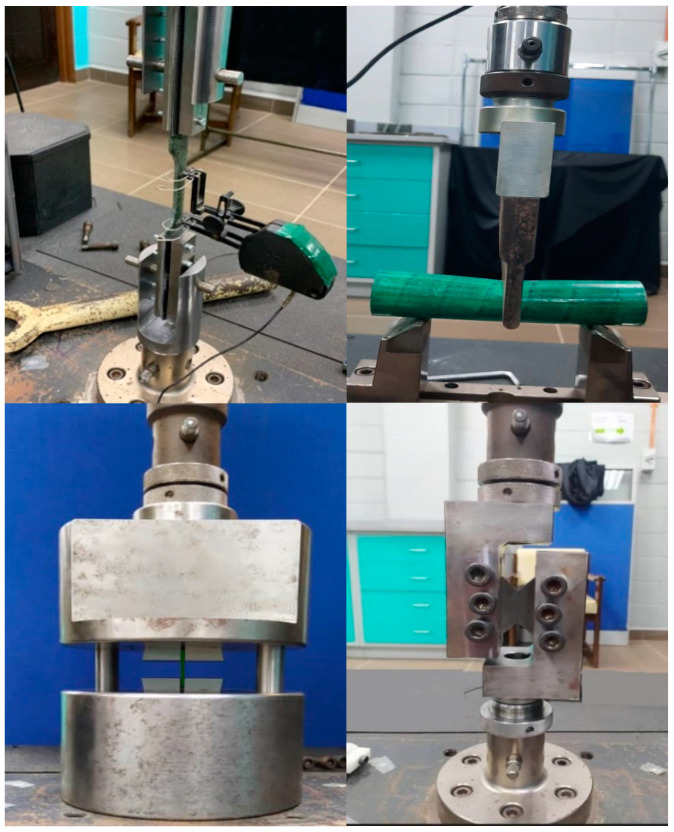
Setup of the experiment for material characterization.

Moreover, to extract material properties of the specimen for use in the FE model, based on ASTM-D3039, ASTM-D695, ASTM D-790, and ASTM D3518/D3518M the test conditions were applied to extract the tensile strength, compressive strength, flexural strength, and in-plane shear strength, respectively. For more detail on data extraction, see [43]. Figure 3 shows the setup of the experiment for the material characterization test. It includes tensile test, compression test, bending flexural test, and shear test. The material properties of hybrid carbon/glass fibre/epoxy unidirectional composite are presented in Table 2.

**Figure 4:**
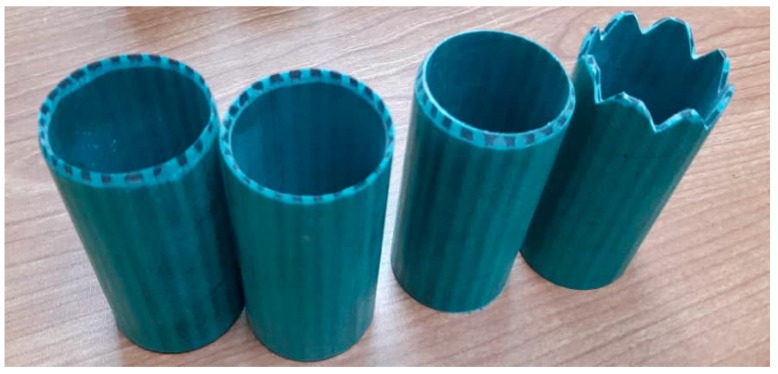
The different chamfered samples as the trigger mechanism.

To evaluate the effect of the trigger mechanism on the energy absorption of composite tubes, four different trigger shapes, which were highlighted from previous works as the best triggers to increase the energy absorption of the structure, were selected. The shapes of different trigger mechanisms included 45° chamfer in (CH-IN), 45° chamfered out (CH-OUT), 45° chamfered in and out (CH-IN/OUT), and a corrugated chamfered (CH-TG) tip. Using a diamond saw machine and a sand grinder machine, different chamfers were applied to the tip of the sample tubes. In this part of the study, the aspect ratio of the tube was considered as L/D = 2 and the loading rate was considered equal to previous tests as 5 mm/min. Figure 4 shows the different chamfered samples used for the axial compression test.

### 2.2. Crashworthiness Indicators

In order to evaluate the crashworthiness of a structure subjected to the impact loading some indicators such as energy absorption (EA), maximum peak load (F_max_), average crush force (F_m_), specific energy absorption (SEA), and crush force efficiency (CFE) should be investigated. In this study, the crash worthiness indicators of composite tubes with different aspect ratios and trigger mechanisms were examined. Energy absorption is the integration of the area under a load-displacement curve, which it is the criterion to demonstrate the stability limit of collapse when comparing different shapes and geometries. The maximum corresponding load is F_max_. The collapse and failure modes in the composite structures are non-symmetrical, hence, it can be predicted that that F_max_ would be different from the first peak load. The average crushing load, F_ave_, is taken from the mean line of collapsing load at every increment of the compression test. Therefore, keeping F_ave_ at the possible highest level increase the area under the curve and subsequently the EA would be at the maximum value. In other words, specimens with higher $F_{\mathrm{ave}}$ have more capacity to absorb energy during compressive deformation, without resulting in instant and catastrophic failure. Energy absorption per volume, SEA, is the crucial indicator to compare the impact resistance of a structure with other different density materials. Improvement of SEA leads to increasing the crash resistance capacity. The other important indicator for assessing crashworthiness performance is CFE. Higher values of this indicator lead to more ideal energy absorption. To read the details of the calculations for the values of the above-mentioned crashworthiness indicators, see [44].

### 2.3. Finite Element Model

**Figure 5:**
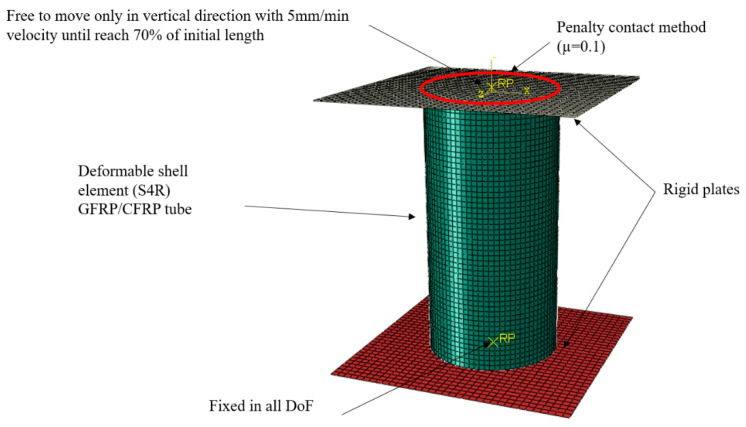
The FE model of the structure under load and the boundary conditions.

An FE model was developed in ABAQUS/Explicit solver. The deformable bodies generated by shell elements (S4R) and two analytical rigid plates were created as the fixed and moving compression machine shoes. A reference point was assigned to the top rigid body and a 5 mm/min velocity was assigned to it. The simulation was stopped when the maximum displacement reached 70% of the initial length. The quality of mesh was verified using two methods. Firstly, the geometrical parameters of the mesh, e.g., aspect ratio and element skew angle were controlled and after obtaining good results a mesh convergence study in a single model was applied and the size of elements was selected for all the case studies [45]. In order to get accurate results and satisfy the quasi-static loading requirements, the balance of energy terms was applied. The total energy should be less than 5% of the subtraction of kinetic energy and internal energy to justify the result of the simulation [46]. The material properties were taken from the experimental test that was reported in Table 1. To model the combination of different fibres in a single matrix, a macro-mechanical approach considering the overall behaviour of specimens was utilized. To study the damage behaviour of structure, Hashin’s damage model was used to model the failure of tubes under axial loading [42]. A surface-to-surface contact penalty method with a 0.1 friction coefficient was applied between the tube end and the rigid plates. In addition, a self-contact to avoid self-penetration of tube walls was applied to the tubes. Figure 5 shows the FE model, applied load, and boundary conditions.

## 3. Results and Discussion

**Figure 6:**
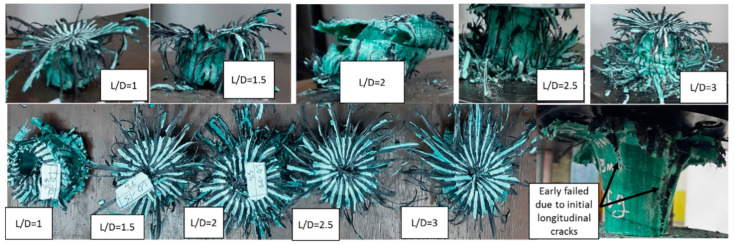
The deformed shape of specimens in the experimental test.

Crashworthiness indicators, i.e., the EA, SEA, F_max_, and CFE were investigated in this study for different aspect ratios. The results of the experimental procedure were used to check the validity of the FE results. The deformed shape of experimental test samples for different aspect ratios is shown in Figure 6. Pre-buckling, buckling, and post-buckling are the three main stages of progressive collapse that can be observed for all samples under axial compressive loading [42]. In the pre-buckling stage, the composite tube remains elastic and has no bending. Gradually, the damage initiates and after reaching the first peak load the buckling stage is initiated and is indicated by a constant or drop in the magnitude of the load. In the buckling mode, the force will be maintained almost at a certain level and every folding and progressive collapse will cause holding of the force at the maximum possible level. The value of CFE indicates the performance of the buckling stage. In the post-buckling mode, the structure has no resistance, and it will collapse with minimum force. This can be named a completely failed structure. The experimental samples show a suitable bonding between carbon fibre, glass fibre, and the epoxy matrix. Unlike other manufacturing methods, such as filament winding and hand layup, the pultrusion method does not experience delamination and debonding in the hoop direction. However, in the fibre direction, some initial cracking was observed and at the onset of the test the crack was propagated, and sudden breakage was detected. This phenomenon was related to the manufacturing process and could be solved by changing some factors in the process, such as the feed rate of the machine and curing time of the specimens.

**Figure 7:**
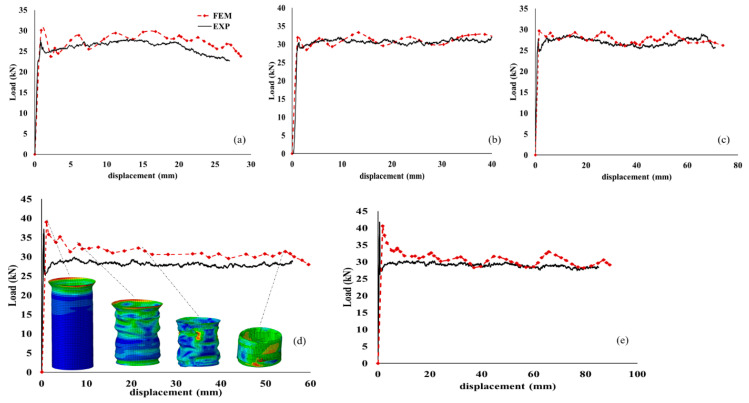
The load-displacement curves for different aspect ratios (**a**) L/D = 1, (**b**) L/D = 1.5, (**c**) L/D = 2, (**d**) L/D = 2.5 and (**e**) L/D = 3 of composite tubes.

Figure 7a–e demonstrates the results of load-displacement curves for different aspect ratios of composite tubes. Only the median curve of different experimental testing is plotted. It can be said that the FE results are in good agreement with the experimental outcomes. Surprisingly, the mean crushing load in all aspect ratios was almost around 30 kN. It can be said that this manufacturing process and the hybridization of a uniform buckling can be achieved. However, in a few samples, some sudden breakages were observed during application of the load.

Table 3 represents the crashworthiness indicators separately, based on the specimen’s aspect ratios. The maximum peak load was 33.58 from L/D-3, whereas the most important parameter in this study was SEA which shows 29.78 from L/D-1.5. The results from Table 2 show that almost all cases have similar results and follow the same trend. Furthermore, the CFE values show that the FE simulation and experimental results have more than 95% crash force efficiency. It means that after the first peak and dropping the force the energy absorption will be maintained with more than 95% of the first peak.

**Figure 8:**
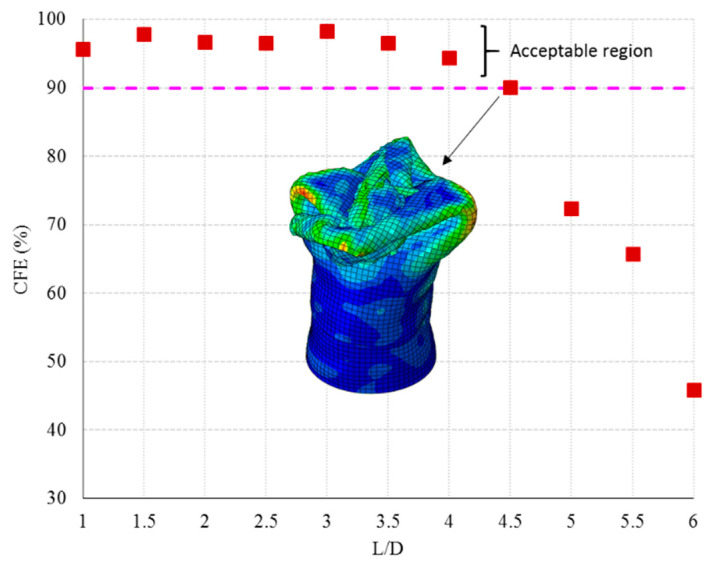
Presentation of L/D influence on the CFE factor.

In fact, in this range of aspect ratios, a critical length was not observed, and to find these factors the test should be continued for higher aspect ratios. To discover the critical length, the FE model was extended for a higher aspect ratio and the critical aspect ratio was found. The criterion to find critical length was calculated by CFE. Figure 8 shows the relationship of the L/D to the CFE. It shows that by increasing the length of the tube the crash force efficiency dropped significantly when the L/D was higher than 4.5. In this case, the progressive collapse cannot be seen and the tube acts as an unsteady collapsed column.

### 3.1. Effect of Trigger Mechanism

**Figure 9:**
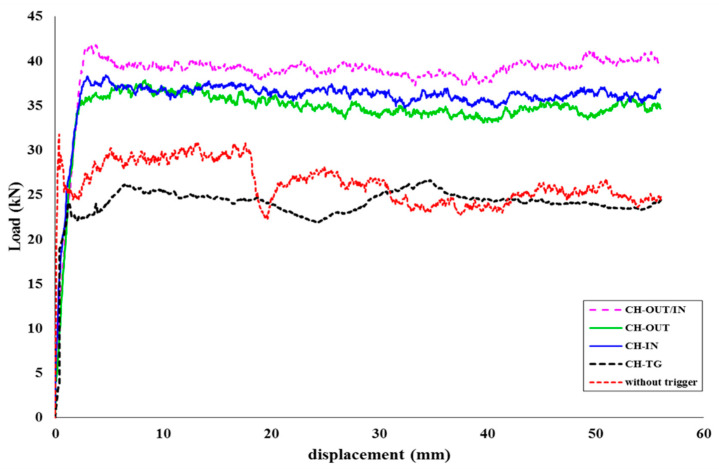
Comparison between triggered mechanisms and normal specimen based on load-displacement curves.

The trigger mechanism provides a region with regulator stress concentration points where structural failure is partial. Therefore, damage can be steadily propagated over the tube length consistently. The crumpling of materials is one of the suitable modes of failure because this process breaks down more fibre and destroys a lot of the matrix. In fact, grinding the fibre and matrix together is the best form of progressive collapse. Four different geometries for the crash initiator mechanism (trigger) were used and the results were taken from the compression testing machine. For each geometry, an FE model was generated, and the experimental testing conditions were applied to the FE model. Figure 9 compares the load-displacement curves of different trigger mechanisms with a normal specimen. This figure shows the deformed specimens after 70% deformation of the initial length. The figure also depicts the deformed shapes of the experimental tests and FE simulations for (CH- OUT), (CH-IN), (CH-IN/OUT), and (CH-TG). The applied continuum damage mechanism (Hashin model) simulated failure modes for each geometry.

**Figure 10:**
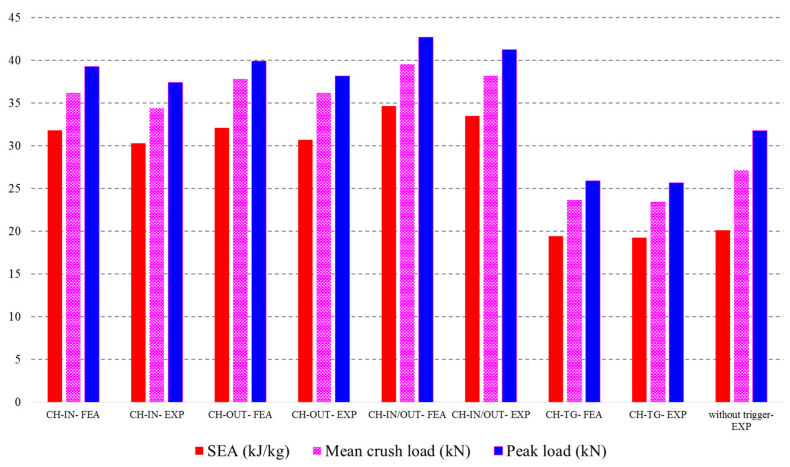
Representation of crashworthiness indicators from FE simulation and experimental data.

Figure 10 compares the results of the experimental test with FE results regarding the three main crashworthiness indicators. The results show that using different trigger mechanisms affects the load-displacement curve significantly. In some cases, the trigger mechanism can create some negative points in terms of energy absorption. The most effective trigger mechanism was the CH-IN/OUT shape and the worst was CH-TG. It can be said that before applying the trigger mechanism to a crash box the geometry should have been justified earlier. In other words, selecting the best geometry can increase the crashworthiness capacity by more than 35%. On the other hand, using inappropriate triggers may reduce the crashworthiness capability by 15%.

The results were compared with a previous study using Kevlar/Aramid fibre to evaluate the energy absorption of a crash box. Kevlar/Aramid is well-known material in impact resistance application and can be considered as the reference for new materials. The results show that the specific energy absorption of the proposed material is 32% lower than Aramid composite [47]. On the other hand, the rate and the cost of production are three times better than Aramid products. Therefore, using the proposed composite tubes in general applications can be suggested as having an appropriate strength with regard to the lower price and production time.

### 3.2. Failure Analysis

Failure observation of flat-end specimens shows some common failure mechanisms. Referring to Figure 6 in all cases, modes I and II and petaling failure modes can be observed. In addition, increasing the length shear failure in the longitudinal direction can be observed. To sum up, the phenomenon in Figure 8 shows that increasing the length of the tube greater than L/D = 4 can cause a sudden break and catastrophic failure. It can be said that finding the critical length for crash resistance is crucial in the design of a hybrid composite tube. In this study, the critical L/D is determined as 4.

**Figure 11:**
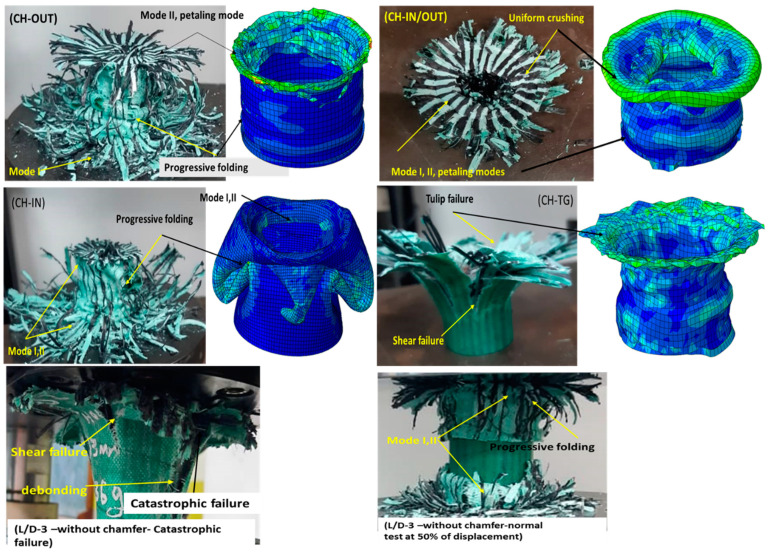
Failure mechanisms of composite tubes with a different chamfering trigger mechanisms.

Using a trigger mechanism has affected the failure modes. For instance, the chamfering trigger at both sides of the tube (CH-IN/OUT) creates failure modes I and II. The results show that using this trigger mechanism has the best absorption among other selected geometries. In terms of failure mechanism and the observation of the failed area, a uniform crumpling area can be seen. Moreover, a petaling failure can be seen at the end of the compression test. The trigger, CH-TG, causes some tearing failure and also shear fracture on the tube walls. The tulip failure can be seen in all samples with CH-TG. This trigger mechanism was the weakest in this study, and using this mechanism brings lower output than samples without a trigger mechanism. In samples CH-IN and CH-OUT, mode I and mode II of fracture as well as petaling failure can be detected. Figure 11 shows different failure modes of different test samples.

The energy absorption of CH-OUT is better than CH-IN, however, the failure mechanism was practically identical. Due to some manufacturing defects caused by increasing the length of tubes, in some cases shear failure and catastrophic failure were observed. However, by controlling production factors this kind of failure can be avoided.

## 4. Conclusions

In this study, the crashworthiness assessment and energy absorption of hybrid glass-carbon fibre/epoxy composite tubes were investigated. The influence of different aspect ratios on the specific energy absorption of the composite tubes was examined. The effects of trigger mechanisms and failure mechanisms were also discussed in this study. The following conclusion is drawn from this study:

The crashworthiness indicators, i.e., EA, SEA, F_max_, and CFE for five tubes with different length aspect ratios were compared and show that by increasing the L/D aspect ratio the SEA was increased slightly, however, finding the critical L/D value shows that the energy absorption drops significantly if the length of the structure passes the critical length. The results of the present study demonstrate that hybrid glass/carbon tubes achieve an average 25.34 kJ/kg specific energy absorption, 1.43 kJ energy absorption, 32.43 kN maximum peak load, and 96.67% crash force efficiency under quasi-static axial loading.

The FE model results were in good agreement with experimental data and the developed model has acceptable accuracy for prediction in further case studies. Moreover, the applied damage model simulated the failure mechanism in all cases. The FE results show that by increasing the L/d ratio, observing shear failure in the longitudinal direction is possible. In this study, the critical length is L/D = 4 and tubes longer than this ratio are exposed to sudden breakage and catastrophic failure.

Four different trigger mechanisms were examined and the CH-IN/OUT shape increase the SEA by more than 35%. The CH-TG mechanism had a negative influence on the energy absorption with the tulip failure mode. CH-IN and CH-OUT increased the SEA by more than 25%. The best trigger mechanism was CH-IN/OUT, with 35 kJ/kg and 40 kN for SEA and F*_max_*, respectively. The failure mode observation of these samples shows mode I, mode II, and pealing failure. Progressive collapse was observed in samples with a chamfered trigger mechanism.

## Figures and Tables

**Table 1:** The general information about the composite tube materials.

Composite Tube Elements	Product Name	Manufacturer	Tensile Strength	Density
Epoxy resin	EPON™ Resin 862	Westlake	76 MPa	1.15 g/cm^3^
hardener	EPIKURE Curing Agent W			
Carbon fibre	PX35	ZOLTEK	4200 MPa	1.2 g/cm^3^
Glass fibre	Glass fibre yarn 0.3 mm	Wee Tee Tong Chemicals	3445 MPa	2.54 g/cm^3^

**Table 2:** The material properties of hybrid carbon/glass fibre/epoxy unidirectional composite.

Symbol	Description	Unit	Value
ρ	Density	Kg/m^3^	1520
$E_{11}$	Module of elasticity	GPa	58
$E_{22,33}$	Module of elasticity	GPa	14.2
$G_{12}$	Shear modules	GPa	11.8
$G_{13,23}$	Shear modules	GPa	0.85
$\vartheta_{12}$	Poisson’s ratio	-	0.29
$\vartheta_{13,23}$	Poisson’s ratio	-	0.14
$X_{T}$	Normal tensile strength	MPa	109
$X_{c}$	Normal compressive strength	MPa	280
$Y_{T}$	Transverse tensile strength	MPa	67
$Y_{C}$	Transverse compressive strength	MPa	190
S	Shear strength	MPa	54
$\varepsilon_{f}$	Fracture strain	-	0.035
$G_{IC}$	Energy release rate	U/mm^2^	20

**Table 3:** The crashworthiness indicators of test specimens with various aspect ratios.

Specimen Label	Crush Length (m)	F_max (Ave)_ (kN)		EA Avg (kJ)		SEA Avg (kJ/kg)		CFE Avg (%)	
		FE	EXP	FE	EXP	FE	EXP	FE	EXP
L/D-1	0.027	29.66	28.20	0.65	0.62	25.1	24.84	95.67	95.27
L/D-1.5	0.04	31.9	32.45	1.21	1.19	29.92	29.78	97.85	95.74
L/D-2	0.056	38.75	27.13	1.1	1.008	20.24	20.16	96.63	95.20
L/D-2.5	0.071	31.2	29.79	1.82	1.76	25.35	25.24	96.56	98.98
L/D-3	0.085	41.98	33.58	2.4	2.22	22.36	22.28	98.31	96.37
